# Persistent Stapedial Artery, Facial Nerve Overhang, and Calcified Stapedius Tendon Leading to Aborted Stapedotomy: An Unusual Middle Ear Anatomy

**DOI:** 10.7759/cureus.81196

**Published:** 2025-03-25

**Authors:** Mebarimon Kharwanlang, Pradipta K Parida, Prity Sharma

**Affiliations:** 1 Otorhinolaryngology - Head and Neck Surgery, All India Institute of Medical Sciences, Bhubaneswar, Bhubaneswar, IND

**Keywords:** calcified stapedius tendon, facial nerve overhang, middle ear anomaly, pediatric hearing loss, persistent stapedial artery, stapedotomy

## Abstract

This report describes a rare medical case of a 16-year-old girl with progressive bilateral hearing loss over nine years. Despite normal external ear anatomy, tests revealed moderate conductive hearing loss. Surgery uncovered an unusual persistent stapedial artery and ossified stapedial tendon with an overhang of the facial nerve over the oval window obscuring it and preventing the planned stapedotomy. Post-surgery, the patient had mild earache but no serious complications and was treated with antibiotics and steroids. She is scheduled for a hearing aid trial. This is the first known report of this exact triad occurring together, posing significant surgical implications. The case underscores the need for detailed preoperative imaging and careful surgical techniques to manage anatomical variations and improve outcomes in middle ear surgeries.

## Introduction

Middle ear conditions that lead to conductive hearing loss include otosclerosis, congenital ossicle fixation, tympanosclerosis, stapedial tendon calcification, and bony bridges to the stapes [1]. Anatomical variations, particularly involving the facial nerve, can further complicate surgical procedures. These include facial canal dehiscence and overhang over the oval window, increasing the risk of iatrogenic facial nerve injury during middle ear surgery [2-4].

Facial nerve anomalies, particularly overhanging or dehiscent segments near the oval window, are commonly encountered in endoscopic middle ear surgery. Their identification is critical, as failure to do so can result in iatrogenic facial palsy. Endoscopic techniques have proven superior to microscopic approaches in identifying such variations early during exploration [5].

Among the rarer anomalies, a persistent stapedial artery (PSA), a congenital vascular remnant, has been reported with a prevalence of 0.02-0.48% and is often associated with stapes agenesis and facial nerve aberrations [6]. While these anomalies may appear in isolation, they share a common embryological origin from the second branchial arch, suggesting that concurrent developmental disruptions may account for their co-occurrence [4,7].

Recent advancements in imaging, such as ultra-high-resolution CT (U-HRCT), enable better detection of subtle middle ear anatomical variants, including facial nerve overhang. Clues such as the angle of the facial nerve stapes and displacement of the promontory can guide surgical planning [8]. However, the triad of PSA, facial nerve overhang, and calcified stapedius tendon has not been previously reported in combination, making this case significant in the context of middle ear surgery.

## Case presentation

Patient Information

A 16-year-old female presented to the Outpatient Department of Otorhinolaryngology at our institute with a chief complaint of bilateral decrease in hearing, which was sudden in onset and progressive in nature over the past nine years and was not associated with trauma, ear discharge, vertigo, or episode of infection. However, it was associated with abnormal ringing sensation in bilateral ears of similar duration with no association with diurnal variation, head movement, headache, nausea, or abnormality in vision. There was no history of previous ear surgery, and the family history was non-contributory. The patient has no other known systemic disease.

This case report follows the principles of the Declaration of Helsinki. Written informed consent was obtained from the patient’s legal guardian for publication of clinical details and accompanying images.

Clinical findings

Examination revealed a normal-appearing external ear, external auditory canal, and tympanic membrane bilaterally.

Tuning fork tests showed Rinne’s test negative bilaterally at 256 Hz, 512 Hz, and 1,024 Hz frequencies. Weber’s test at 512 Hz demonstrated lateralisation to the left ear.

**Figure 1:**
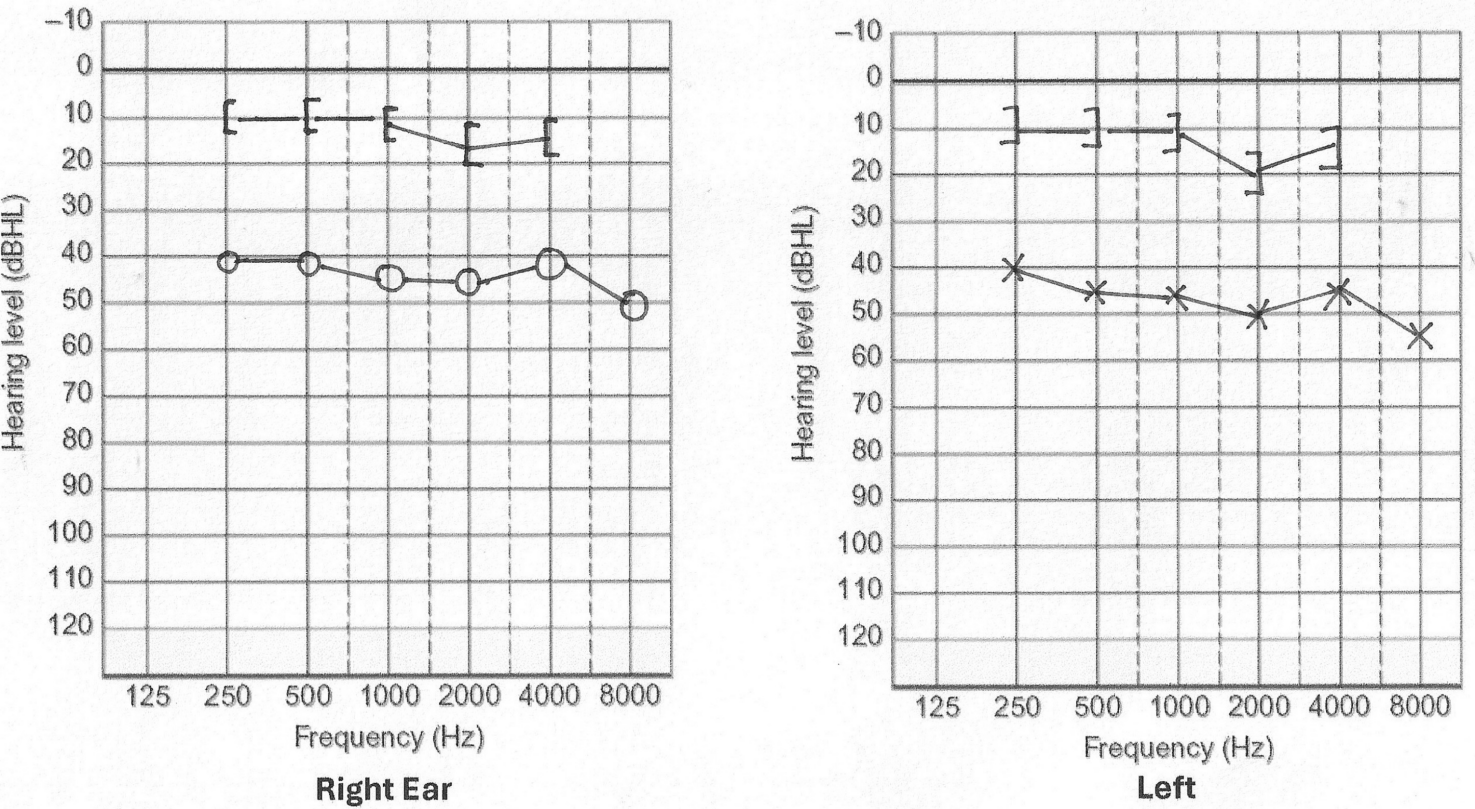
Audiometry screening

The audiogram showed bilateral moderate conductive hearing loss in the right ear, with a pure tone average of 41d BHL air conduction (AC) and 15d BHL bone conduction (BC) over the frequencies of 500Hz, 1,000 Hz, 2,000 Hz, and 4,000 Hz, whereas the left ear had a pure tone average of 50 dBHL AC and 18 dBHL BC over the frequencies of 500 Hz, 1,000 Hz, 2,000 Hz, and 4,000 Hz (Figure 1).

Immittance test showed a right ear external canal volume of 1.10 cm3, gradient of 54 with 7 Decapascal of pressure, and an “A” type of waveform. The left ear canal volume was 0.88 cm3, gradient was 44 with 16 Decapascal of pressure, and there was an “As” type of waveform.

Speech audiometry revealed a speech reception threshold of 50 dB HL bilaterally, with 100% word recognition scores in both ears.

Radiological findings

**Figure 2:**
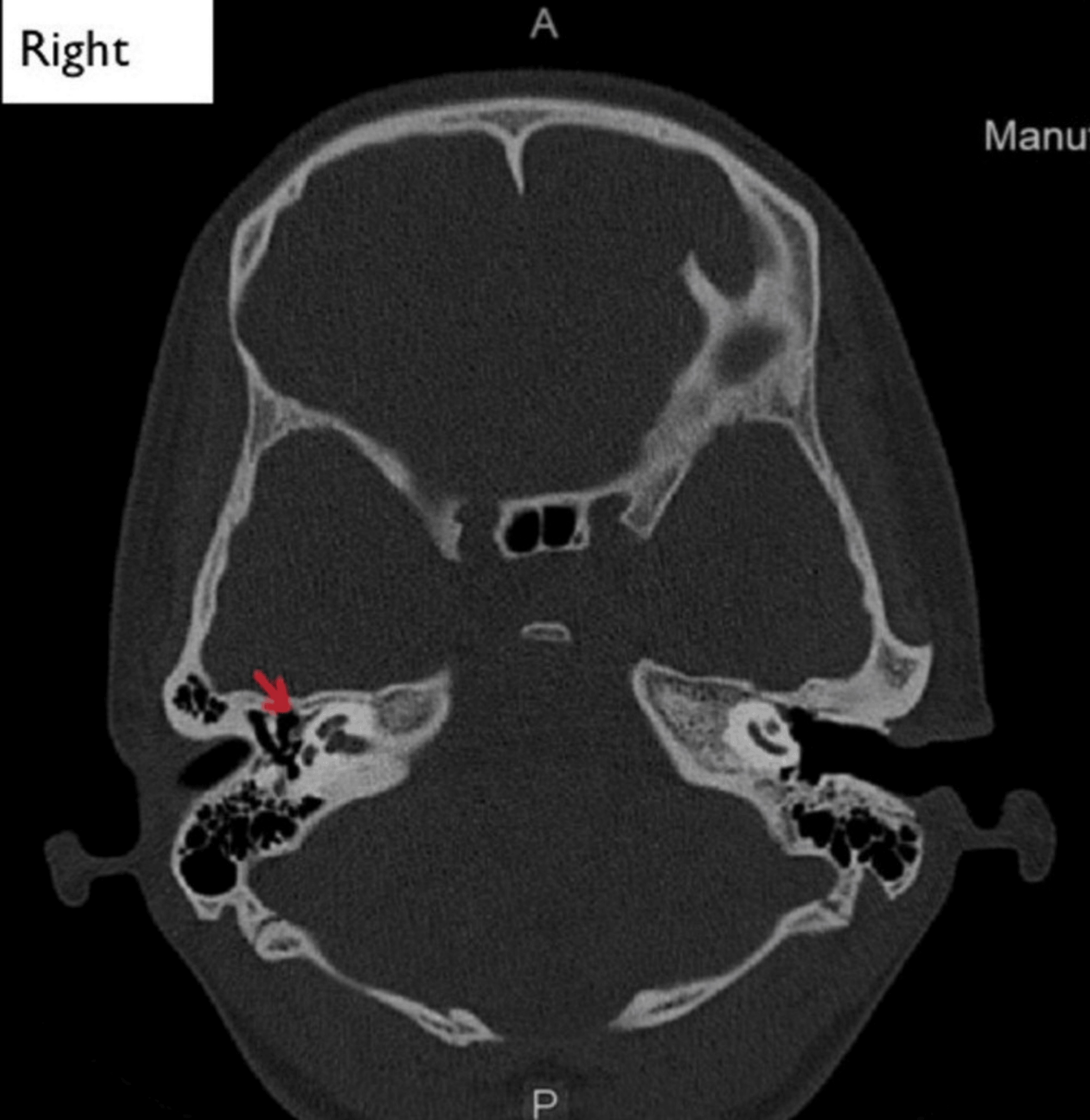
Coronal section of high-resolution computed tomography of the right temporal bone. The red arrow points to the oval window niche and facial nerve.

**Figure 3:**
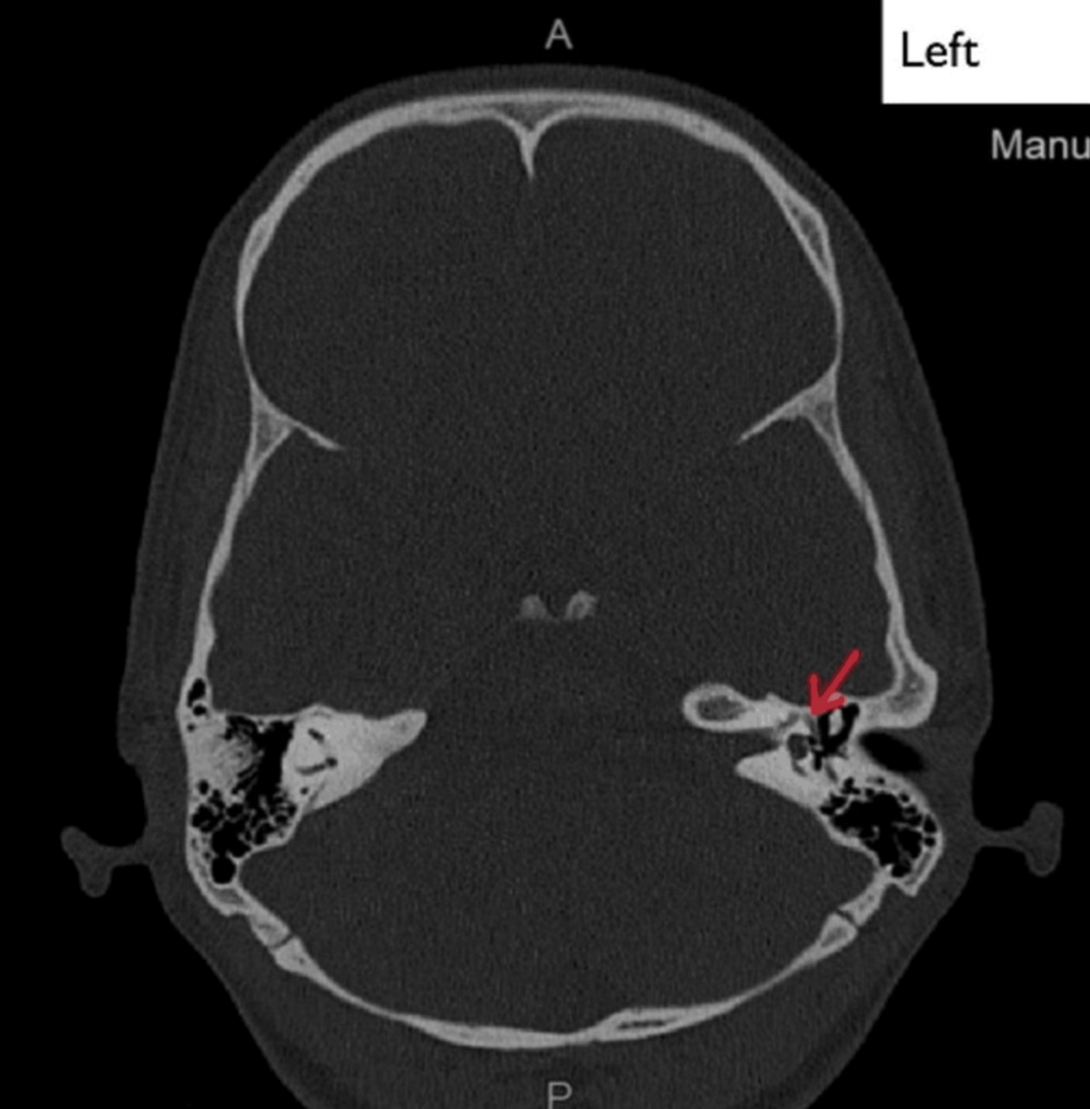
Coronal section of high-resolution computed tomography of the left temporal bone. The red arrow points to the oval window niche and facial nerve.

High-resolution computed tomography (HRCT) of the temporal bone detected no other abnormalities in the middle and inner ear (Figures 2, 3).

Intervention

**Figure 4:**
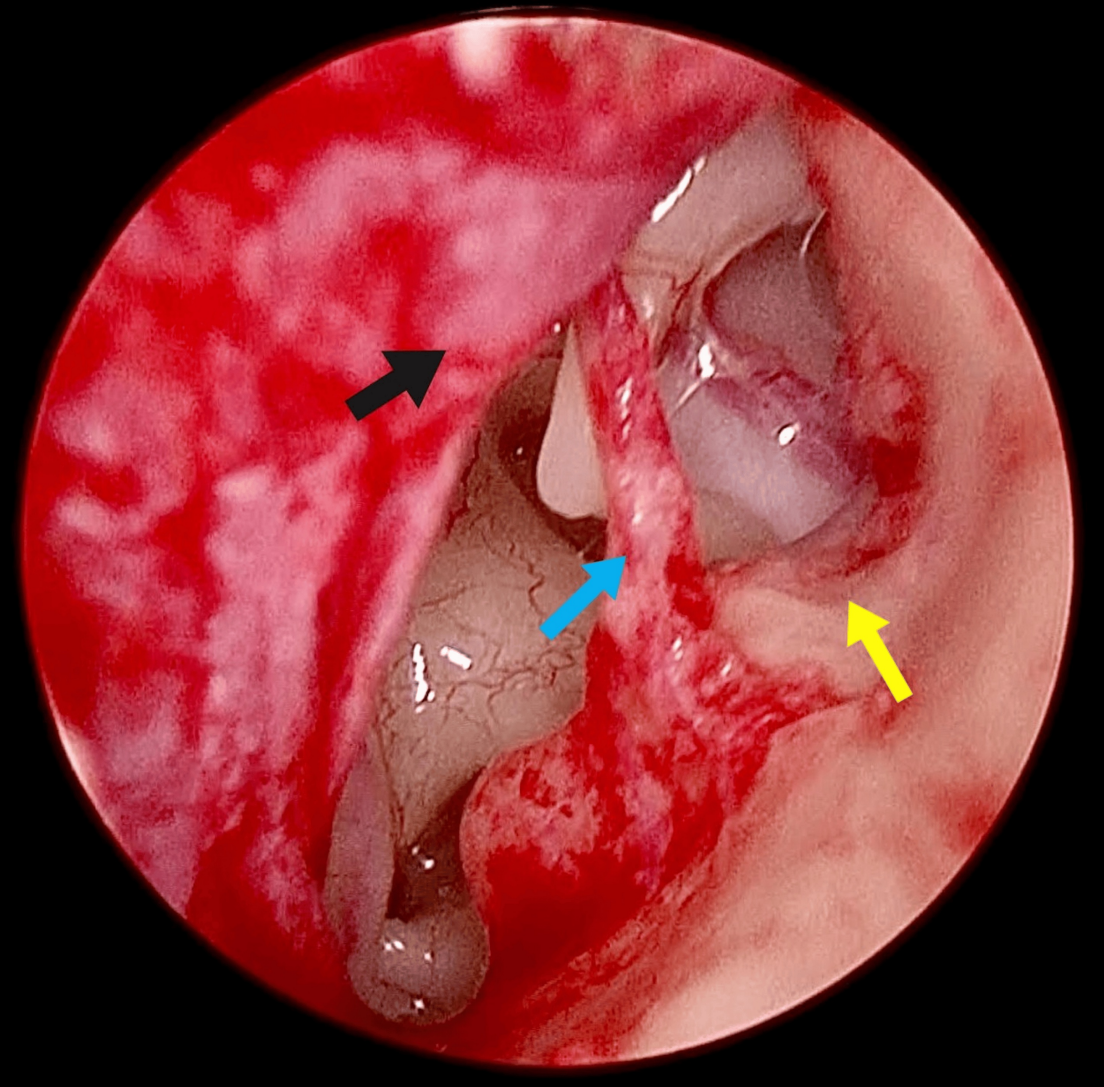
Narrow extra auditory canal. Posterior bony wall overhang (yellow arrow) was curetted with a micro curette. Chorda tympani nerve (blue arrow) and tympanomeatal flap (black arrow) are also visualized.

A clinical diagnosis of left ossicular fixation was suspected, and an exploratory tympanotomy was conducted via the transcanal endoscopic approach under local anesthesia. The medial end of the extra auditory canal wall near the posterior part of the annulus was narrow, and the footplate of stapes was not visualized; hence, posterior wall overhang curetting with a micro curette for wider exposure and transposition of the chorda tympani nerve were performed (Figure 4).

**Figure 5:**
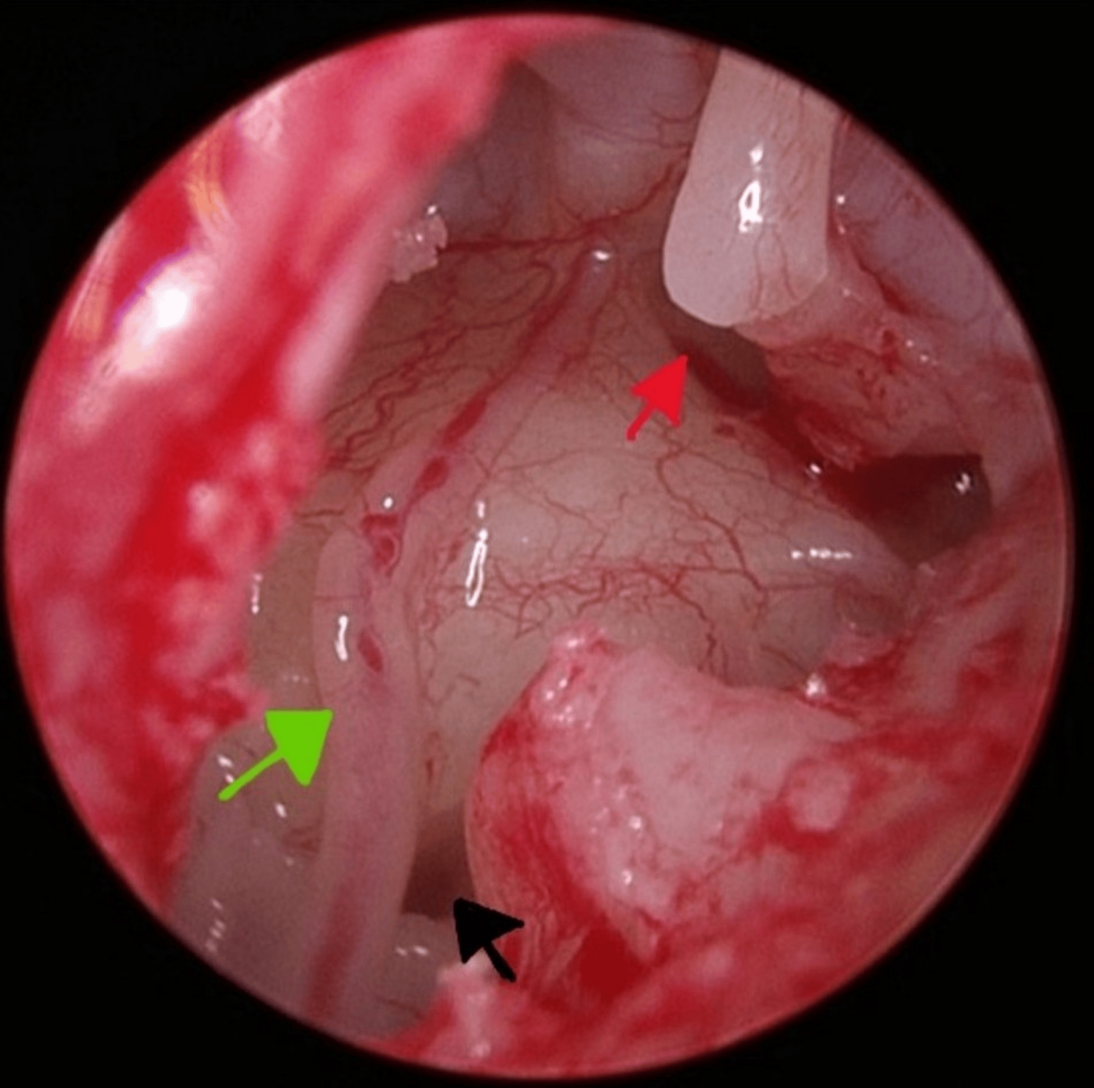
Persistent stapedial artery over the promontory (green arrow), round window (black arrow), and incudostapedial joint (red arrow).

Upon lifting the tympanomeatal flap, a PSA was observed running across the promontory (Figure 5).

**Figure 6:**
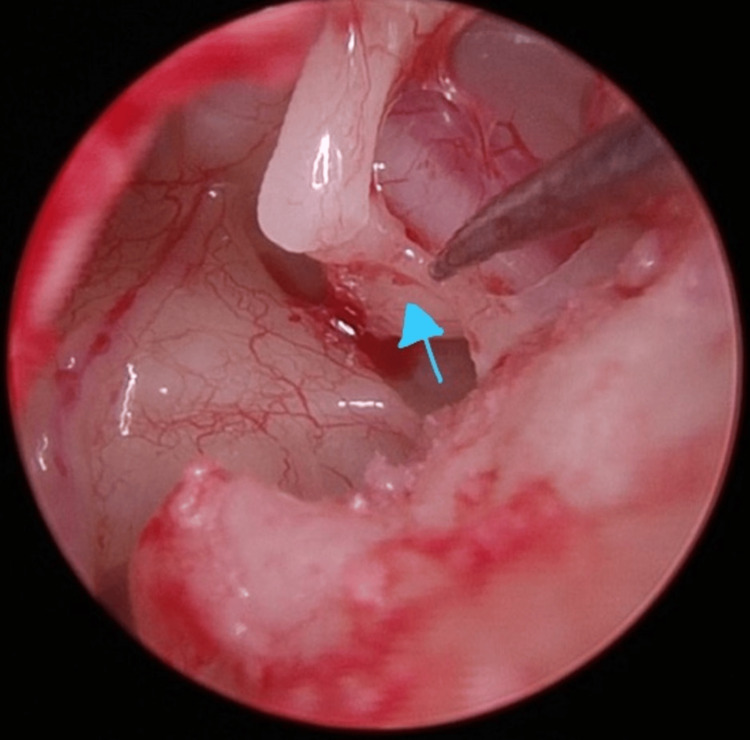
Calcified stapedial tendon (blue arrow).

No ossicles were absent or abnormally shaped. Malleus and incus were mobile, but the stapes was fixed. The stapedial tendon was seen to be calcified (Figure 6).

**Figure 7:**
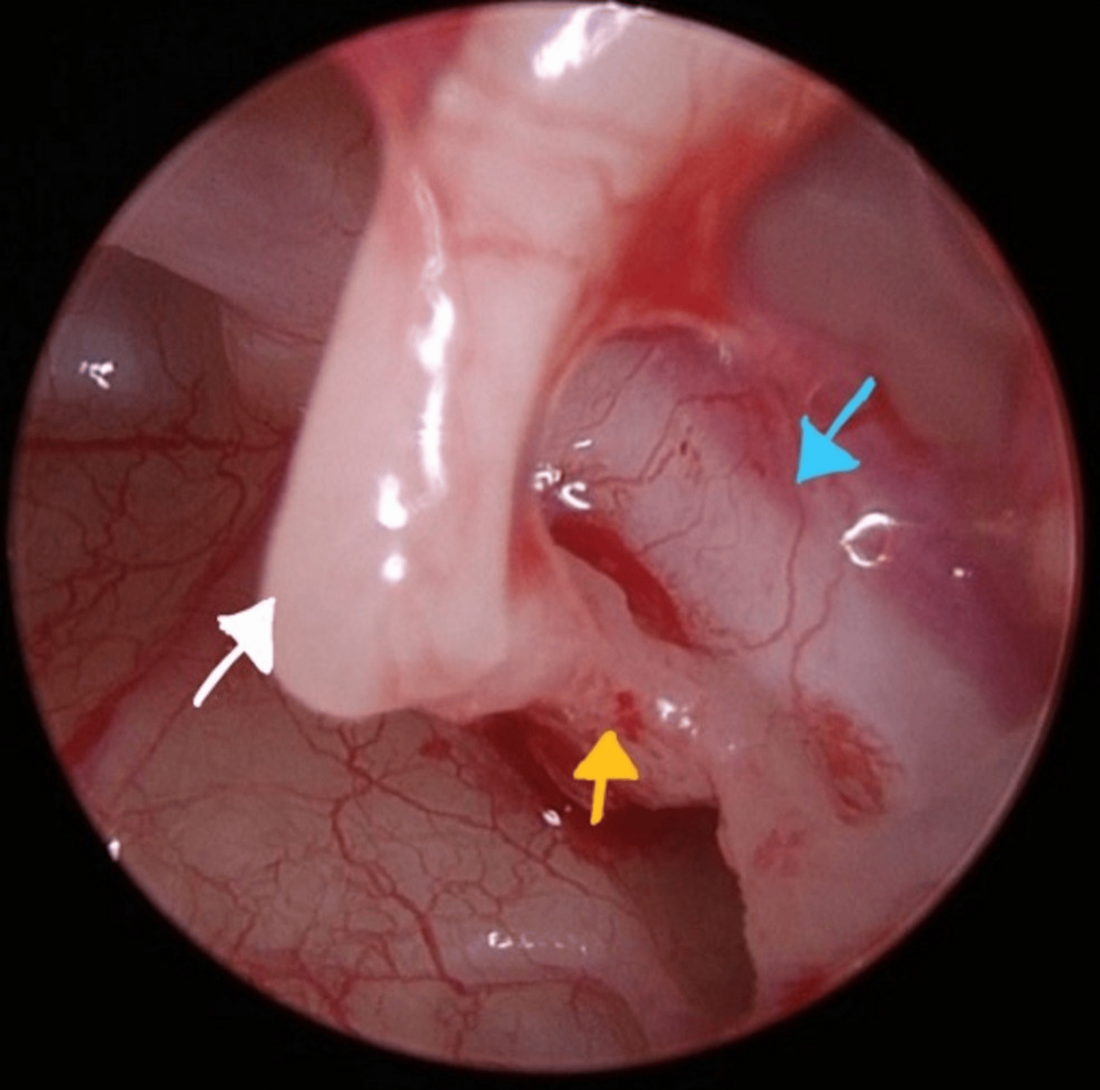
Long process of the incus (white arrow), posterior crura of the stapes (yellow arrow), and tympanic segment facial nerve overhanging obscuring the round window (blue arrow).

There was an overhang of the tympanic segment of the facial nerve, with adherence of the nerve to the anterior and posterior crura of the stapes and non-visibility of the stapes footplate (Figure 7).

**Figure 8:**
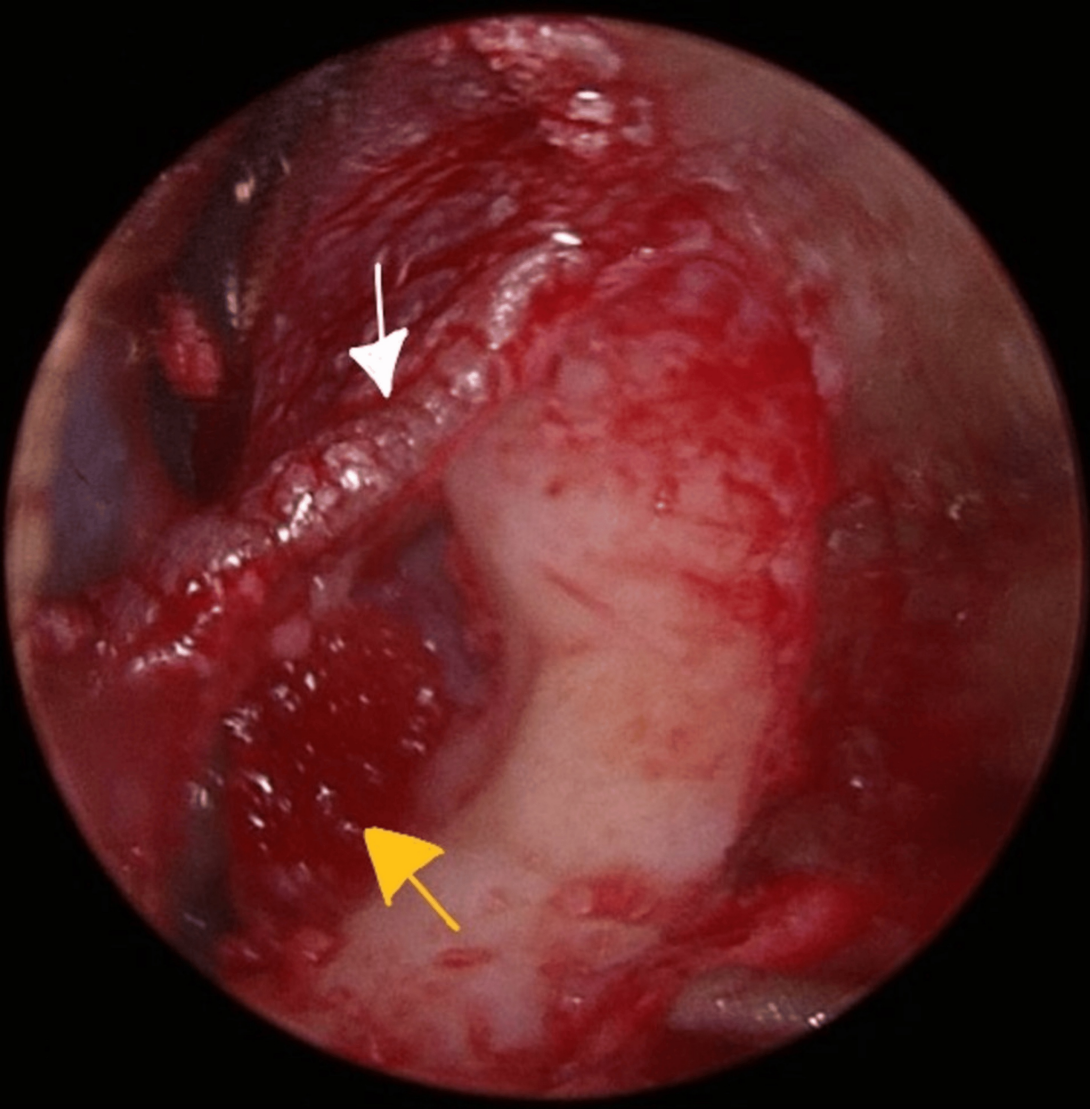
Tympanomeatal flap (white arrow) gel foam placed in the middle ear (yellow arrow).

Stapedotomy, which was earlier planned to restore hearing, was aborted given the abnormal findings. The middle ear was packed with gel foam packing (Figure 8).

The tympanomeatal flap was reposited, and medicated (ciprofloxacin+ dexamethasone ear drops) gel foam was used for the packing of the external canal.

Outcomes

The immediate post-operative period was uneventful. The patient had mild earache in the post-operative period, with no facial nerve palsy or nystagmus. Post-operative Weber’s test was performed on the patient, which showed lateralization on the left side, ie., same as preoperative Weber’s test. The patient was managed conservatively with antibiotics (1.2g intravenous amoxyclav thrice daily), analgesics (1g intravenous paracetamol infusion thrice daily), steroid (8mg intravenous dexamethasone twice daily), and oral multivitamin tablet and was discharged after 24 hours on oral medications as per treatment protocols.

Given the high surgical risk posed by the observed anomalies, stapedotomy was deferred. The patient is planned for a hearing aid trial.

## Discussion

The coexistence of a PSA, facial nerve overhang with adherence to the stapes, and a calcified stapedius tendon in our patient represents a unique triad of congenital middle ear anomalies, not previously described in combination. All three structures arise from the second branchial (pharyngeal) arch, and their simultaneous maldevelopment suggests a common embryological disruption during the fourth to tenth week of gestation [4,7,9].

PSA results from failure of regression of the embryonic stapedial artery and is often associated with stapes or facial nerve anomalies. Gheorghe et al. described a similar constellation involving PSA with oval window atresia and stapes agenesis, managed with cochleostomy and TORP [6]. In contrast, our case had an intact but fixed stapes, which, along with the proximity of the PSA and adherent facial nerve, made surgical manipulation unsafe. Furthermore, Goderie et al. reviewed surgical strategies for PSA and emphasized the importance of preoperative identification to avoid hemorrhage or neurovascular injury [10].

The facial nerve anomaly in our case - its overhang and adherence to the stapes crura - posed a major surgical hazard. Baxter and later Thomas et al. reported that dehiscence of the facial canal at the oval window is not uncommon and significantly increases the risk of iatrogenic injury during middle ear procedures [3,11]. Magliulo et al. further emphasized the importance of intraoperative facial nerve monitoring (FNM) in complex stapedotomies to mitigate this risk [12]. In our case, even with endoscopic visualization, the facial nerve’s close relationship to the ossicular chain prevented safe surgical access. In similar future scenarios, use of real-time FNM may assist in preserving facial nerve integrity.

The ossified stapedius tendon further contributed to stapes immobility, a rare but reported cause of non-footplate stapes fixation. Although Larem et al. reported a case involving stapedial suprastructure fixation with mobile footplate, the clinical takeaway is similar: inadequate identification of fixation sites can lead to floating footplate or sensorineural damage [13]. Hence, our team opted against any further manipulation once the triad was visualized.

Our case reinforces findings from previous reports, such as that by Gheorghe et al. [6], who described PSA associated with stapes agenesis and facial nerve overhang. However, the presence of PSA with stapes fixation due to tendon ossification and facial nerve adherence-without agenesis-sets our case apart, highlighting a new variant combination.

As highlighted by Soloperto et al. [5], intraoperative recognition of PSA and facial nerve variants is essential to prevent hemorrhage and neurologic complications. Our intraoperative decision to abort the stapedotomy aligns with this cautious approach, prioritizing patient safety.

Emerging surgical options also include endovascular occlusion of PSA, which has been successfully reported by Bloch et al. as a preoperative measure to mitigate intraoperative vascular complications [14]. Though still under investigation, this method may offer future therapeutic potential for cases where PSA obstructs stapes access.

The use of a transcanal endoscopic approach in our case was pivotal, allowing us to visualize the PSA and overhanging facial nerve. Transcanal endoscopic approach has been shown to offer superior access and clarity in narrow middle ear spaces, especially where anatomical variations are present [2]. It also reduces the need for extensive canaloplasty and facilitates safer decision-making intraoperatively.

These anomalies were not distinctly visible on preoperative HRCT, underlining the limitations of conventional imaging. U-HRCT, as shown by Zhang et al. and Xu et al., offers superior resolution and diagnostic consistency, detecting lesions <1 mm with high sensitivity and reducing observer variability [8,15]. Such modalities may have preoperatively revealed the full extent of ossicular and nerve anomalies in our case.

Unusual variants like those described by Kim and Wilson, including third mobile windows or cochlear apex defects, also highlight the breadth of anatomical possibilities that can present as conductive hearing loss [16]. Moreover, a population-based study by Pauli et al. correlated congenital ear anomalies with paternal age, suggesting broader genetic or epigenetic underpinnings that may explain such rare findings [17].

Given the combination of PSA, facial nerve adherence, and tendon ossification, we recommend a structured decision-making algorithm when evaluating similar challenging cases:

• Step 1: Advanced imaging with U-HRCT and radiologic consultation

• Step 2: Identification of PSA, facial nerve course, and ossicular mobility

• Step 3: Consider the use of FNM and endoscopic access planning

• Step 4: If PSA is obstructive, evaluate for endovascular embolization [14]

• Step 5: Avoid surgery if anatomy precludes safe manipulation-opt for non-surgical rehabilitation [18].

## Conclusions

This case highlights a rare and surgically significant combination of congenital middle ear anomalies: PSA, facial nerve overhang with adherence to the stapes, and a calcified stapedius tendon. The presence of this unique triad, likely sharing a common embryological origin from the second branchial arch, made standard stapedotomy unsafe and necessitated abortion of the procedure. The use of transcanal endoscopic ear surgery allowed detailed visualization; however, the presence of vascular and neural anomalies warranted surgical restraint. Intraoperative abortion was not a failure but a strategic decision to prevent catastrophic bleeding or facial nerve damage.

Advancements such as FNM, U-HRCT, and even preoperative endovascular management may offer safer solutions in the future. Ultimately, a flexible, anatomy-based approach that prioritizes patient safety, aided by multidisciplinary planning, is crucial when managing complex congenital middle ear anomalies.
